# Declines in muscle protein synthesis account for short‐term muscle disuse atrophy in humans in the absence of increased muscle protein breakdown

**DOI:** 10.1002/jcsm.13005

**Published:** 2022-05-23

**Authors:** Matthew S. Brook, Tanner Stokes, Stefan H.M. Gorissen, Joseph J. Bass, Chris McGlory, Jessica Cegielski, Daniel J. Wilkinson, Bethan E. Phillips, Ken Smith, Stuart M. Phillips, Philip J. Atherton

**Affiliations:** ^1^ MRC‐Versus Arthritis Centre for Musculoskeletal Ageing Research and NIHR Nottingham BRC, Centre Of Metabolism, Ageing and Physiology (COMAP), School of Medicine University of Nottingham Derby UK; ^2^ School of Life Sciences University of Nottingham Nottingham UK; ^3^ Department of Kinesiology McMaster University Hamilton ON Canada; ^4^ School of Kinesiology and Health Studies Queen's University Kingston ON Canada

**Keywords:** Immobilization, Atrophy, Muscle, Protein synthesis, Protein breakdown

## Abstract

**Background:**

We determined the short‐term (i.e. 4 days) impacts of disuse atrophy in relation to muscle protein turnover [acute fasted‐fed muscle protein synthesis (MPS)/muscle protein breakdown (MPB) and integrated MPS/estimated MPB].

**Methods:**

Healthy men (*N* = 9, 22 ± 2 years, body mass index 24 ± 3 kg m^−2^) underwent 4 day unilateral leg immobilization. *Vastus lateralis* (VL) muscle thickness (MT) and extensor strength and thigh lean mass (TLM) were measured. Bilateral VL muscle biopsies were collected on Day 4 at *t* = −120, 0, 90, and 180 min to determine integrated MPS, estimated MPB, acute fasted‐fed MPS (l‐[ring‐^13^C_6_]‐phe), and acute fasted tracer decay rate representative of MPB (l‐[^15^N]‐phe and l‐[^2^H_8_]‐phe). Protein turnover cell signalling was measured by immunoblotting.

**Results:**

Immobilization decreased TLM [pre: 7477 ± 1196 g, post: 7352 ± 1209 g (*P* < 0.01)], MT [pre: 2.67 ± 0.50 cm, post: 2.55 ± 0.51 cm (*P* < 0.05)], and strength [pre: 260 ± 43 N m, post: 229 ± 37 N m (*P* < 0.05)] with no change in control legs. Integrated MPS decreased in immob vs. control legs [control: 1.55 ± 0.21% day^−1^, immob: 1.29 ± 0.17% day^−1^ (*P* < 0.01)], while tracer decay rate (i.e. MPB) (control: 0.02 ± 0.006, immob: 0.015 ± 0.015) and fractional breakdown rate (FBR) remained unchanged [control: 1.44 ± 0.51% day^−1^, immob: 1.73 ± 0.35% day^−1^ (*P* = 0.21)]. Changes in MT correlated with those in MPS but not FBR. MPS increased in the control leg following feeding [fasted: 0.043 ± 0.012% h^−1^, fed: 0.065 ± 0.017% h^−1^ (*P* < 0.05)] but not in immob [fasted: 0.034 ± 0.014% h^−1^, fed: 0.049 ± 0.023% h^−1^ (*P* = 0.09)]. There were no changes in markers of MPB with immob (*P* > 0.05).

**Conclusions:**

Human skeletal muscle disuse atrophy is driven by declines in MPS, not increases in MPB. Pro‐anabolic therapies to mitigate disuse atrophy would likely be more effective than therapies aimed at attenuating protein degradation.

## Introduction

Periods of skeletal muscle atrophy due to general ill‐health, immobility, sedentary behaviours, trauma, disease, or ageing punctuate the age‐related declines in skeletal muscle mass.^1^ While the aetiology of muscle atrophy depends on the underlying cause, a major component of all‐cause muscle atrophy is physical inactivity. Inactivity may occur slowly, through sedentarism/immobility, or more rapidly due to enforced bed rest or limb casting after trauma or elective surgery.^2^, ^3^, ^4^ It follows that a significant body of research has, and continues to focus upon, the mechanisms of muscle atrophy and mitigation strategies.^5^ In terms of the temporal nature of muscle atrophy upon immobilization, there is growing evidence of rapidity over short time frames (<1 week).^4^, ^6^ The average UK NHS hospital stay is ~4.5 days (Source: NHS HES 2018–2019) and time off work due to flu related illness ~3 days.^7^ However, the mechanisms of atrophy during short‐term immobilization remain unclear in humans.^8^

Muscle mass is regulated by the balance between muscle protein synthesis (MPS) and muscle protein breakdown (MPB) rates. These rates exist in dynamic equilibrium resulting from fasted‐fed cycles whereby dietary protein intake replenishes amino acids released from muscle for extra‐muscular processes in between meals.^1^ Theoretically, muscle disuse atrophy could be driven by declines in MPS, increases in MPB, or both. Early studies using stable isotopically labelled amino acid (AA) tracer infusions have shown that declines in post‐absorptive MPS rates are a consistent feature of human disuse atrophy.^9^, ^10^ Similarly, MPS responses to protein intake during immobilization are also blunted, albeit through mechanisms not easily explained by classical AA sensing.^11^ Nonetheless, it is clear that blunted rates of fasted and fed MPS are key players in human disuse atrophy.^9^, ^12^

In contrast to MPS, the role of MPB in human disuse atrophy remains poorly defined, despite decades of work generated in animal models suggesting that MPB is a major driver of disuse atrophy. The lack of MPB data in humans is in part due to the technical challenges of quantifying MPB *in vivo*. Indeed, while MPS is readily measurable using ‘direct incorporation (into muscle biopsy)’ tracer techniques, such a gold standard does not exist for MPB. The arterio‐venous (A‐V) balance technique is one approach to quantify tracer rate of appearance/disappearance, although this requires limb—typically femoral—venous cannulation and is reliant upon assumptions and accurate and timely measures of blood flow.^13^ Another approach is the ‘pulse‐chase’ technique, capitalizing on the ratio of dilution between two tracers over time.^14^ Of the few studies that have quantified MPB, one showed no effect on MPB (using A‐V balance technique) after 14 day bed rest in the face of decreased MPS and whole‐body protein synthesis.^15^ Moreover, short‐term studies measuring dynamic MPB are lacking, despite reports of heightened static biomarkers of MPB in the early phase of disuse atrophy.^4^, ^16^, ^17^ Therefore, a major gap remains in our understanding of the relative roles of MPS/MPB and the technical challenges to quantifying these dynamic processes over an entire disuse period.

In the present study, we aimed to address each of these knowledge gaps by determining the short‐term (4 days) impact of disuse atrophy using the knee brace immobilization model^9^ in humans in relation to: MPS (acute fasted‐fed via l‐[ring‐^13^C_6_]‐phenylalanine and cumulative MPS via D_2_O), acute pulse‐chase tracer decay rate representative of MPB, and estimated 4d FBR, in addition to regulatory pathways of MPS/MPB. We hypothesized that declines in acute fasted and fed, and thus cumulative MPS would account for short‐term disuse atrophy. We further hypothesized that MPB would not be playing a major role in human disuse atrophy.

## Methods

### Participant characteristics

Nine young, healthy male participants^11^ (mean ± SD: age, 22 ± 2 years; body mass index, 24 ± 3 kg m^−2^) volunteered to take part in this study. A sample size of nine in each group was determined to have a power of 95% and a significance level of 5%, based on previously published data showing a lower post‐prandial stimulation of MPS in the immobilized vs. control limb (0.02 ± 0.007 vs. 0.044 ± 0.010% h^−1^).^17^ Participants were initially screened by medical questionnaire, with exclusions for history of any neuromuscular disorder or muscle/bone wasting disease, acute or chronic metabolic, respiratory or cardiovascular disorder, or any other signs of ill health. All participants were performing activities of daily living or recreation upon entry to the study but were not routinely undertaking heavy, structured exercise. Participants did not use tobacco‐containing products or consume excessive alcohol (>21 units per week). The experimental procedures were approved by the Hamilton Integrated Research Ethics Board (HiREB #2192) and conformed to the Declaration of Helsinki. Written informed consent was obtained from all participants prior to their participation.

### Experimental procedures

**Figure 1:**
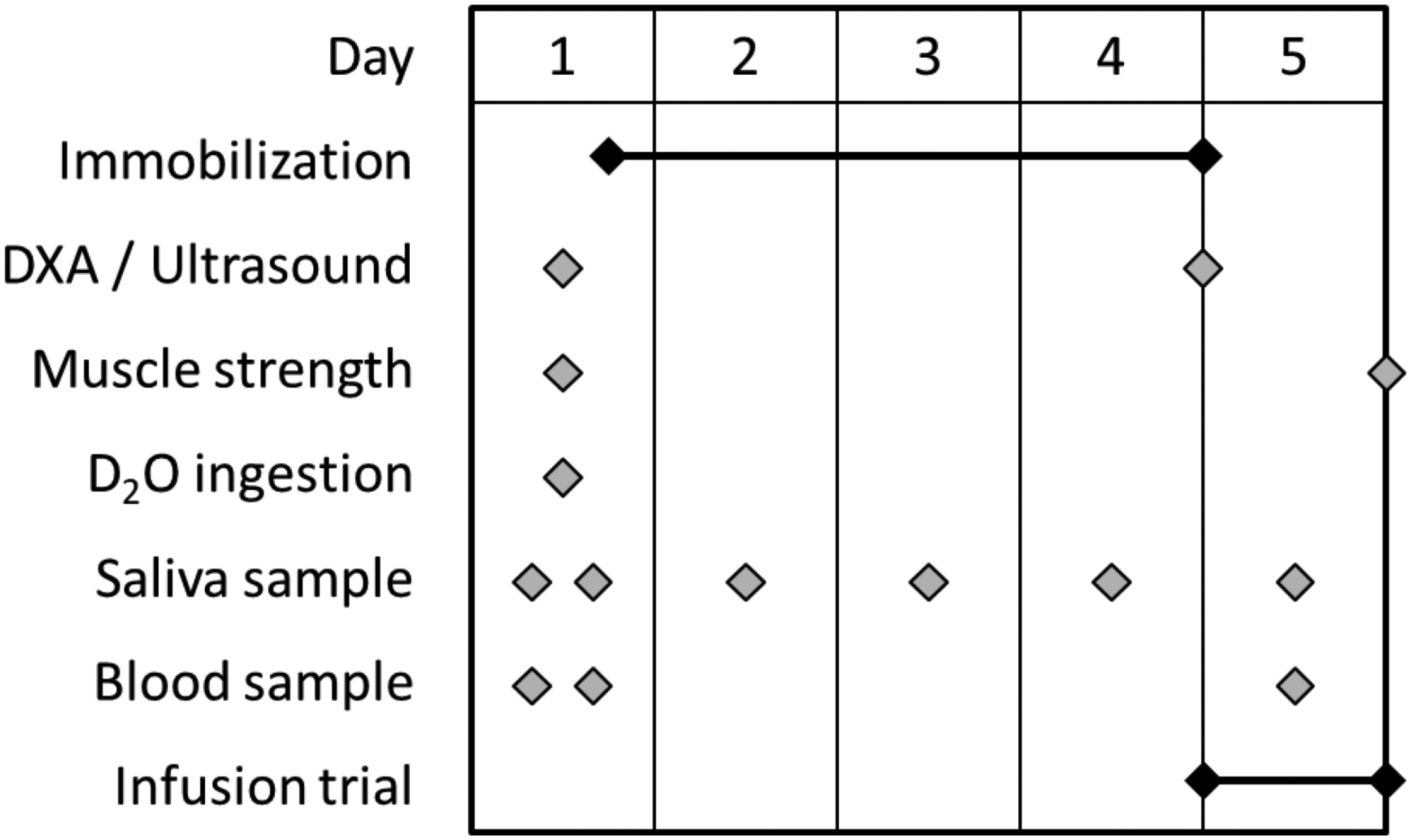
Schematic representation of study design.

This study involved a bilateral leg protocol, with one leg randomly assigned to be immobilized and the contralateral limb used as a co‐temporal control. Upon inclusion, participants were asked to visit the laboratory on two separate occasions, both of which followed an overnight fast (*Figure* 1). On the first visit, participants had the thigh lean mass of both legs measured using dual‐energy X‐ray absorptiometry (DXA) (Lunar iDXA; GE Medical Systems, Mississauga, ON), before ingesting a bolus dose of D_2_O (3 mL kg^−1^) for the measurement of integrated MPS rates. A saliva and blood sample was obtained prior to and 2 h following D_2_O consumption for measurement of deuterium enrichment in body water. *Vastus lateralis* (VL) muscle thickness (MT) was assessed using B‐mode ultrasonography (Vivid Q, GE Medical Systems, Horten, Norway). During the first assessment, a mark was made with permanent marker 50% of the distance between the greater trochanter of the femur and the lateral epicondyle of the knee, identified by palpation. A thick layer of acoustic gel was applied on the leg at the area of measurement, and care was taken to avoid depressing the muscle belly during image acquisition. The test–retest intraclass correlation coefficient for VL MT was 0.96. Maximal voluntary isometric contraction torque (MVC) of each leg was assessed using Biodex dynamometer (Biodex System 3; Biodex Medical Systems, Shirley, New York). The test–retest intraclass correlation coefficient for Biodex measures was 0.92. Thereafter, a knee brace (X‐Act ROM; DonJoy, Dallas, TX, USA) was fitted to the immobilization randomized leg and fixed at an angle of 60° of knee flexion. Participants continued to wear the knee brace as described for a duration of 3 days, collecting a saliva sample each day, before returning to the laboratory for their second visit exactly 4 days after the first visit. Upon arrival for Visit 2, participants had their knee brace removed, DXA thigh lean mass measured, ultrasound scan, and a further saliva sample was obtained. Participants were transported between stations in a wheelchair to prevent weight bearing in the immobilized leg prior to undergoing the infusion trial described below.

### Infusion protocol

**Figure 2:**
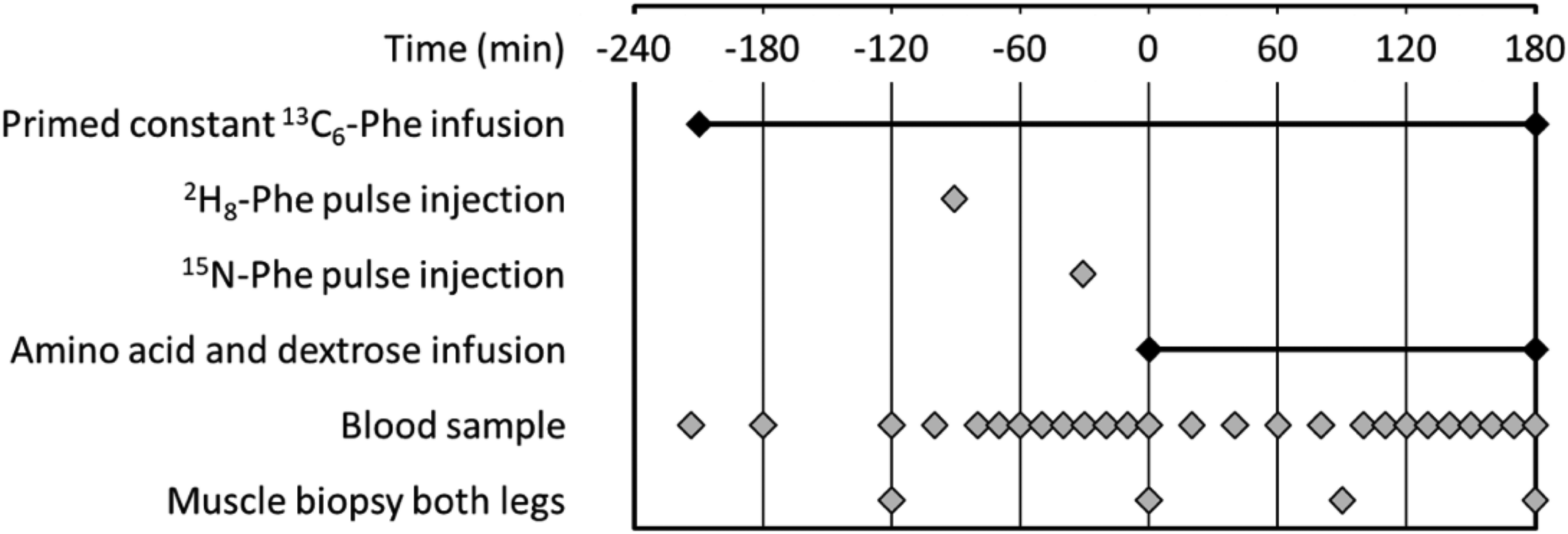
Schematic representation of infusion trial.

Participants reported to the laboratory in the overnight fasting state (at least 10 h fasted). The knee brace was removed, and participants remained in a supine position on a laboratory bed for the entire duration of the infusion trial. A catheter was placed in an antecubital vein for AA and dextrose infusions. A second catheter was inserted into an antecubital vein of the contralateral arm and placed in a heated blanket for arterialized blood sampling. After obtaining a baseline blood sample (*t* = −210 min), a primed constant infusion of l‐[ring‐^13^C_6_]‐phenylalanine (0.4 mg kg^−1^ prime; 0.6 mg kg h^−1^ infusion) was initiated for the assessment of MPS. Additional blood samples were collected regularly throughout (*Figure* 2). At *t* = −120 min, a saliva sample was collected, and the first muscle biopsies were taken from both the immobilized and control leg for measurement of integrated MPS. Further muscle biopsies were collected from both legs at *t* = 0 min to determine basal MPS based on l‐[ring‐^13^C_6_]‐phenylalanine incorporation. Pulse injections of l‐[^2^H_8_]‐phenylalanine at *t* = −90 min (5.53 mg kg^−1^) and l‐[^15^N]‐phenylalanine at *t* = −30 min (5.4 mg kg^−1^) for the determination of MPB rates (represented by tracer decay rate over time) were applied in the fasted state, calculated from muscle biopsies at *t* = 0 min. After collecting the muscle biopsies, primed constant infusions of AAs (34 mg kg^−1^ prime; 102 mg kg h^−1^ infusion; PRIMENE 10%, Baxter Corporation, Mississauga, Ontario, Canada) and dextrose (blood glucose clamped at 7.0–7.5 mM) were started to mimic a post‐prandial state for the remainder of the infusion trial. During the post‐prandial state, the l‐[ring‐^13^C_6_]‐phenylalanine infusion rate was increased to 0.86 mg kg h^−1^ to account for unlabelled phenylalanine entering the free AA pool and prevent dilution of the precursor pool. At *t* = 90 and *t* = 180 min, muscle biopsies were collected from the immobilized and control legs. After the final biopsies, participants were provided with juice and a snack, the dextrose infusion was gradually decreased, and other infusions were stopped. Plasma glucose was monitored for another 30 min in order to prevent hypoglycaemia. Blood samples were collected in EDTA containing tubes and centrifuged at 1800*g* for 10 min at 4°C. Aliquots of plasma were frozen in liquid nitrogen and stored at −20°C. Biopsies were collected from the middle region of the *m. vastus lateralis*, approximately 15 cm above the patella and 3 cm below entry through the fascia, using the percutaneous needle biopsy technique. Muscle samples were dissected carefully, freed from any visible non‐muscle material, immediately frozen in liquid nitrogen, and stored at −80°C until further analysis. A schematic representation of the infusion trial can be seen in *Figure* 2.

### Glucose clamp

During the infusion trial, starting at *t* = 0 min, a glucose clamp was initiated to mimic a post‐prandial state. Dextrose (25%) was infused according to a priming infusion protocol adapted from DeFronzo *et al*.^18^ for the first 14 min until a steady‐state blood glucose concentration of 7.0–7.5 mM was obtained. Steady‐state blood glucose was maintained by measuring blood glucose concentrations every 5 min and adjusting the infusion rate accordingly. Blood glucose concentrations over the course of the dextrose infusion were measured using the YSI STAT 2300 glucose analyser. At the end of the infusion trial, participants consumed juice and a snack while the dextrose infusion rate was gradually decreased in order to prevent hypoglycaemia. Blood glucose was monitored for another 30 min.

### Body water, muscle protein bound alanine, and intracellular free amino acid enrichments

Body water enrichment was measured as previously described.^19^ Briefly, saliva was heated in a vial at 100°C, then cooled rapidly on ice and the condensate transferred to a clean vial ready for analysis. Deuterium enrichment was measured on a high‐temperature conversion elemental analyser connected to an isotope ratio mass spectrometer (TC/EA‐IRMS Thermo Finnigan, Hemel Hempstead, UK). For isolation of myofibrillar and sarcoplasmic fractions, 30–50 mg of muscle was used. Muscle samples were homogenized in ice‐cold homogenization buffer [50 mM Tris–HCl (pH 7.4), 50 mM NaF, 10 mM ␤‐glycerophosphate disodium salt, 1 mM EDTA, 1 mM EGTA, and 1 mM activated Na_3_VO_4_ (all from Sigma‐Aldrich)] and a complete protease inhibitor cocktail tablet (Roche, West Sussex, UK) at 10 μL μg^−1^ tissue. Homogenates were rotated for 10 min, and the supernatant (sarcoplasmic fraction) was collected by centrifugation at 1000*g* for 15 min at 4°C. The pellet was resuspended in 500 μL mitochondrial extraction buffer, dounce homogenized, and the supernatant removed after centrifugation at 1000*g* for 5 min at 4°C. The myofibrillar pellet was solubilized in 0.3 M NaOH and separated from the insoluble collagen by centrifugation, and the myofibrillar protein was precipitated in 1 M perchloric acid. The myofibrillar protein was then precipitated, hydrolysed in 0.1 M HCL for 24 h, and the free AAs purified and derivatized as their *n*‐methoxycarbonyl methyl esters. Incorporation of deuterium into protein bound alanine was determined by gas chromatography‐pyrolysis‐isotope ratio mass spectrometry (GC‐pyrolysis‐IRMS, Delta V Advantage; Thermo Finnigan, Hemel Hempstead, UK) alongside a standard curve of known DL‐alanine‐2,3,3,3‐D_4_ enrichment to validate measurement accuracy. MPS, fractional growth rate (FGR), and fractional breakdown rate (FBR) were calculated as previously described.^20^ The incorporation of l‐[ring‐^13^C_6_]‐phenylalanine was determined by gas chromatography‐combustion‐isotope ratio mass spectrometry (Delta plus XP; Thermo Fisher Scientific, Hemel Hempstead, United Kingdom) with muscle intracellular l‐[ring‐^13^C_6_]‐phenylalanine, l‐[^15^N]‐phenylalanine, and l‐[^2^H_8_]‐phenylalanine enrichment measured by gas chromatography‐tandem mass spectrometry (TSQ 8000; Thermo Scientific) following precipitation of the sarcoplasmic fraction and purification of the supernatant using Dowex H^+^ resin as described above, with AAs converted to their m*ethoxycarbonyl ethyl esters*. The fractional synthesis rate of the myofibrillar proteins was calculated using the precursor‐product equation as previously described.^21^ The tracer decay rate or MPB was calculated using pulse‐chase methods (*n* = 5). From muscle biopsies collected at *t* = 0 min, the enrichment of l‐[^15^N]‐phenylalanine and l‐[^2^H_8_]‐phenylalanine was calculated. Pulse injections of l‐[^15^N]‐phenylalanine and l‐[^2^H_8_]‐phenylalanine were administered at *t* = −90 min and *t* = −30 min respectively, the tracer dilution within the intracellular pools enables a decay rate representing MPB to be calculated.^22^, ^23^, ^24^ The exponential tracer decay rate was determined from the difference in intracellular enrichment of l‐[^15^N]‐phenylalanine and l‐[^2^H_8_]‐phenylalanine at *t* = 0 biopsy over 1 h.

### Immunoblotting for muscle signalling pathway activity

Immunoblotting was performed as previously described^25^ using the sarcoplasmic fraction isolated from myofibrillar preparation (*n* = 8). Sarcoplasmic protein concentrations were analysed using a NanoDrop ND1000 spectrophotometer (NanoDrop Technologies, Inc., Wilmington, DE‐US) and sample concentrations adjusted to 1 μg μL^−1^ in 3× Laemmli buffer to ensure equivalent protein loading in pre‐cast 12% Bis‐Tris Criterion XT gels (Bio‐Rad, Hemel Hempstead, UK) of 10 μg/lane. Samples were separated electrophoretically at 200 V for 1 h, followed by transfer of proteins to PVDF membrane at 100 V for 45 min and subsequent blocking in 2.5% non‐fat milk in Tris‐buffered saline/Tween 20 (TBST) for 1 h. Membranes were incubated in primary antibodies (1:2000 dilution in 2.5% BSA in TBST) overnight at 4°C; *p*‐mTOR Ser 2448 (#2972), *p*‐4E‐BP1 Thr 37/46 (#2855), *p*‐eEF2 Thr 56 (#2331), *p*‐AKT Ser 473 (#4060), *p*‐rpS6 Ser 235/236 (#2211), *p*‐eIF4E Ser 209 (#9741), *p*‐eIF4B Ser 422 (#3591), Ubiquitin (1:1000; #3933, New England Biolabs, Hertfordshire, UK), Calpain (1:1000; ab3589, Abcam, Cambridge, UK), and MAFbx (1:1000; #AP2041, ECM Biosciences, Versailles, KY, USA). Membranes were subsequently washed and incubated in HRP conjugated anti‐rabbit secondary antibody (#7074, New England Biolabs, Hertfordshire, UK; 1:2000 in 2.5% BSA in TBST) at ambient temperature for 1 h, before exposure to chemiluminescent HRP Substrate (Millipore Corporation, Billerica, MA, USA) for 5 min. Bands were quantified by Chemidoc XRS (Bio‐Rad, Hertfordshire, UK). All signals were within the linear range of detection; loading was corrected to Coomassie [34]. Fed (*t* = 90 min) data were normalized to control mean and transformed using *Y* = (log(1 + *Y*)).

### Statistical analyses

Data are presented as means ± standard deviation (SD) or as individual data points with paired samples connected by a line. Data were tested for normal distribution using a Kolmogorov–Smirnov test. DXA lean mass, VL MT, MVC, and acute MPS were analysed using a repeated measures two‐way ANOVA. Immunoblotting values were normalized to control and transformed using *Y* = (log(1 + *Y*)) prior to analysis using a repeated measures two‐way ANOVA. Integrated MPS, FGR, FBR, and tracer decay rate were analysed using a paired *t*‐test. All data analysis was performed using GraphPad Prism (GraphPad Software Inc., San Diego, CA); correlations were assessed using Pearson's product moment correlation coefficient. Cohen's *d* effect size (*d*) was calculated for paired samples. The alpha level of significance was set at *P* < 0.05.

## Results

### Muscle mass, *vastus lateralis* thickness, and strength

**Figure 3:**
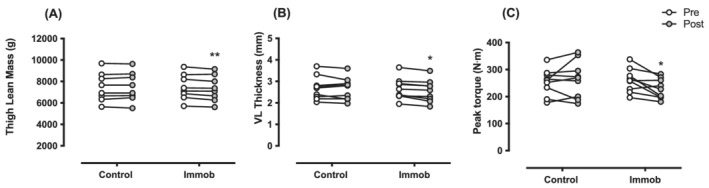
Thigh lean mass (*A*), vastus lateralis (VL) muscle thickness (*B*), and maximal voluntary isometric contraction torque (*C*) of the control and immobilized (immob) leg pre and post 4 days of single leg immobilization in health young men. **P < 0.01, *P < 0.05.

At baseline, there was no difference in TLM, VL MT, or strength between legs. Over the 4 day study period, the control leg showed no change in TLM [pre: 7476 ± 1341 g, post: 7501 ± 1349 g (*d* = 0.26)], VL MT [pre: 2.68 ± 0.54 cm, post: 2.65 ± 0.52 cm (*d* = 0.23)] or MVC [pre: 253 ± 48 N m^−1^, post: 264 ± 68 N m^−1^ (*d* = 0.36)]. The immobilized leg showed a decrease from baseline in TLM [pre: 7477 ± 1196 g, post: 7352 ± 1209 g, (*P* < 0.01, *d* = 1.1) (main effect of interaction: *P* = 0.01, time: *P* = 0.06, intervention: *P* = 0.09), *Figure* 3A], VL MT [pre: 2.67 ± 0.50 cm, post: 2.55 ± 0.51 cm, (*P* < 0.05, *d* = 1.45) (main effect of interaction: *P* = 0.16, time: *P* = 0.01, intervention *P* = 0.8), *Figure* 3B], and MVC [pre: 260 ± 43 N m^−1^, post: 229 ± 37 N m^−1^, (*P* < 0.05, *d* = 0.93) (main effect of interaction: *P* = 0.02, time: *P* = 0.22, intervention *P* = 0.54), *Figure* 3C].

### Integrated muscle protein synthesis, fractional growth and breakdown rates

**Figure 4:**
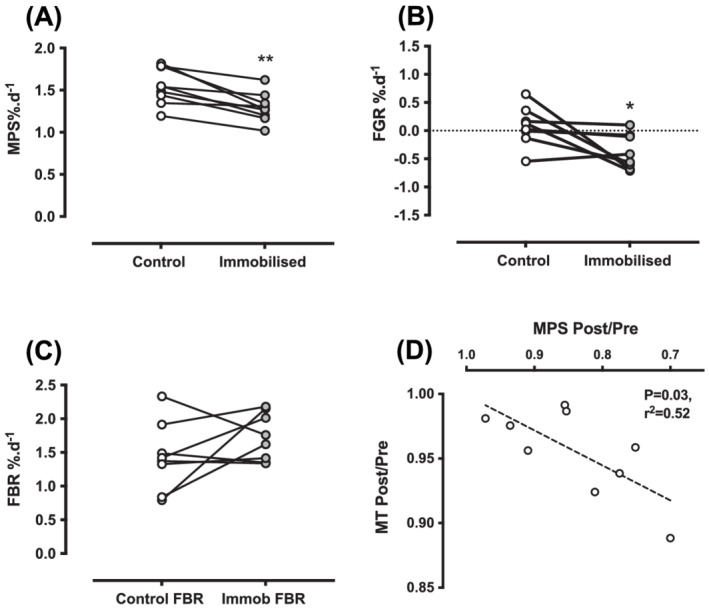
Over the 4 day study period in control and immobilized legs (*A*) muscle protein synthesis in % day^−1^, (*B*) fractional growth rate in % day^−1^, (*C*) fractional breakdown rate in % day^−1^, and (*D*) correlation between the change in MPS and change in VL MT. **P < 0.01, *P < 0.05.

Over the study period, 4 day integrated MPS was decreased in the immobilized vs. the control leg [control: 1.55 ± 0.21% day^−1^, immob: 1.29 ± 0.17% day^−1^, (*P* < 0.01 *d* = 1.35), *Figure* 4A]. Similarly, the FGR was decreased in the immobilized leg [control: 0.08 ± 0.34% day^−1^, immob: −0.38 ± 0.31% day^−1^, (*P* < 0.05, *d* = 0.77) *Figure* 4B], while the FBR remained unchanged [control: 1.44 ± 0.51% day^−1^, immob: 1.73 ± 0.35% day^−1^, (*P* = 0.21 *d* = 0.39), *Figure* 4C]. The change in MT was correlated with the change in MPS (*P* = 0.03, *r* ^2^ = 0.52, *Figure* 4D) and was not correlated with the change in FBR (*P* = 0.17, *r* ^2^ = 0.29, not shown).

### Acute muscle protein turnover

**Figure 5:**
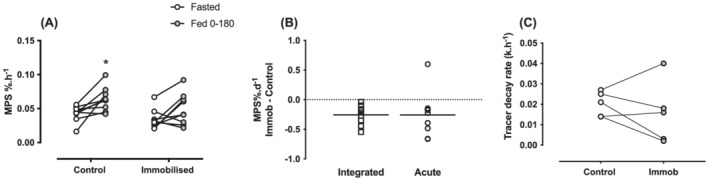
(*A*) Acute fasted and fed MPS rates in % h^−1^ in control and immobilized legs. (*B*) The absolute change in integrated or acute muscle protein synthesis in % day^−1^ (assuming two‐thirds of the day is spent fasted and one‐third fed). (*C*) Acute fasted tracer decay rate (k h^−1^ value). *P < 0.05.

**Figure 6:**
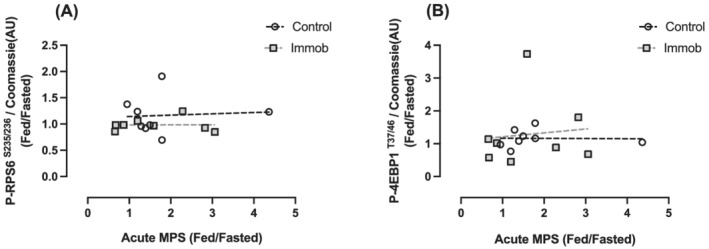
Correlation between change in fasted to fed muscle protein synthesis vs. (*A*) change in fasted to fed p‐rpS6 and (*B*) change in in fasted to fed p‐4E‐BP1.

**Figure 7:**
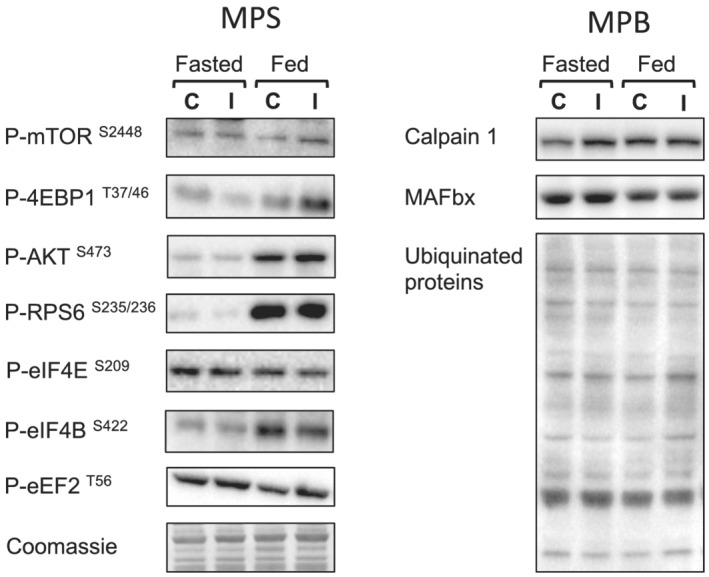
Representative immunoblots for muscle signalling pathway activity.

After 4 days of immobilization, there was an overall trend for decreased MPS, yet fasted and fed rates of acute MPS remained unchanged (*Figure* 5A) (main effect of interaction: *P* = 0.53, time: *P* = 0.008, intervention *P* = 0.07). However, only the control leg increased MPS in response to feeding [fasted: 0.043 ± 0.012% h^−1^, fed: 0.065 ± 0.017% h^−1^, (*P* < 0.05, *d* = 1.38)] with no change in the immobilized leg [fasted: 0.034 ± 0.014% h^−1^, fed: 0.049 ± 0.023% h^−1^ (*P* = 0.09, *d* = 0.90) *Figure* 5A]. The absolute decrease in integrated or acute MPS was similar [integrated: −0.25 ± 0.16% day^−1^, acute: −0.26 ± 0.38% day^−1^ (assuming two‐thirds of the day is spent fasted and one‐third fed) (*d* = 0.004), *Figure* 5B]. The acute fasted tracer decay rate representing MPB did not differ between control and immobilization legs [control: 0.02 ± 0.006, immob: 0.015 ± 0.015, (*d* = 0.89) *Figure* 5C]. The change in MPS was not correlated with either the change in rpS6 or 4E‐BP1 phosphorylation from fasted to fed states (*Figure* 6A,B). Overall, both anabolic and catabolic markers were similar between control and immob, with no effect of immobilization on markers of MPB (*Table* 1) (*Figure* 7).

**Table 1:** Muscle anabolic and catabolic signalling in control and immobilized legs

		Fasted	Fed	
P‐mTOR^S2448^	C	0.60 ± 0.45	0.61 ± 0.24	Time = 0.89 Intervention = 0.69 Interaction = 0.84
	I	0.56 ± 0.48	0.52 ± 0.26	
P‐4EBP1^T37/46^	C	0.67 ± 0.21	0.72 ± 0.17	Time = 0.73 Intervention = 0.09 Interaction = 0.54
	I	0.84 ± 0.29	0.84 ± 0.24	
P‐AKT^S473^	C	0.67 ± 0.21	1.02 ± 0.48	Time = 0.006 Intervention = 0.94 Interaction = 0.56
	I	0.60 ± 0.21	1.12 ± 0.59*	
P‐RPS6^S235/236^	C	0.52 ± 0.60	1.78 ± 0.52*	Time = 0.001 Intervention = 0.81 Interaction = 0.77
	I	0.57 ± 0.47	1.62 ± 0.96*	
P‐eIF4E^S209^	C	0.66 ± 0.27	0.66 ± 0.22	Time = 0.21 Intervention = 0.85 Interaction = 0.15
	I	0.78 ± 0.28	0.59 ± 0.19	
P‐eIF4B^S422^	C	0.69 ± 0.13	0.88 ± 0.42	Time = 0.05 Intervention = 0.46 Interaction = 0.88
	I	0.59 ± 0.28	0.77 ± 0.36	
P‐eEF2^T56^	C	0.68 ± 0.17	0.65 ± 0.16	Time = 0.88 Tntervention = 0.76 Interaction = 0.36
	I	0.66 ± 0.12	0.71 ± 0.11	
Calpain 1	C	0.69 ± 0.12	0.74 ± 0.11	Time = 0.52 Intervention = 0.17 Interaction = 0.30
	I	0.78 ± 0.10	0.77 ± 0.09	
MAFbx	C	0.63 ± 0.36	0.76 ± 0.23	Time = 0.45 Intervention = 0.83 Interaction = 0.28
	I	0.74 ± 0.43	0.72 ± 0.32	
Ubiquitin	C	0.69 ± 0.09	0.76 ± 0.16	Time = 0.92 Intervention = 0.20 Interaction = 0.15
	I	0.70 ± 0.08	0.64 ± 0.09	

Data were normalized to control mean and transformed using *Y* = (log(1 + *Y*)).

* *P* < 0.05.

## Discussion

Muscle disuse through lifestyle changes, injury, illness, or short‐term hospitalization results in loss of skeletal muscle mass and strength.^2^, ^3^, ^26^ This muscle wasting is particularly evident with long‐term immobilization,^27^ yet there is growing evidence that muscle mass loss proceeds most rapidly during the early stages of disuse.^4^, ^28^ Substantial muscle wasting occurs after 4 days of immobilization,^29^ likely having critical consequences for many individuals undergoing short‐term inactivity through acute hospitalization [~4.5 days (Source: NHS HES 2018–2019)] or illness (~3 days^7^). On this basis, it has been speculated that repeated short‐term periods of immobilization may accelerate an older individual's trajectory to frailty.^30^ Nevertheless, the proteostasis underpinnings of human disuse muscle atrophy and strength declines remain unclear, leaving us without effective countermeasures. Here, we discovered that reduction in muscle size and strength following 4 days of immobilization was driven by declines in MPS and no change in D_2_O‐derived FBR. In addition, declines in MT correlated with change in MPS but not FBR. Feeding‐induced hyperaminoacidemia showed a main effect of feeding with no significant interaction. However, there was a more robust and expected stimulation of MPS in the control leg, that was ablated in the immobilized leg, highlighting anabolic resistance as key element in muscle disuse atrophy. Finally, we observed no change in acute tracer decay, representative of MPB, or intracellular markers of MPB. Our results point to the prominent role of declines in MPS and not elevated proteolysis as the dominant mechanism of early muscle disuse atrophy.

There are numerous models of human muscle disuse atrophy (e.g. bed rest, casting, unilateral lower‐limb suspension) that vary in mobility restrictions and clinical relevance, yet they all share common outcomes of decreased muscle mass and strength.^5^ For instance, reductions in strength of 9% have been reported with 5 days of leg casting^4^ and 25% with 14 days of unilateral lower‐limb suspension.^10^ Here, we report losses of ~11% in just 4 days, adding to previous literature of rapid losses of muscle strength.^29^ Loss of strength is accompanied by muscle mass declines, with losses in muscle volume of 1.7% by 2 days and 6.7% by 7 days reported via MRI.^28^ In addition to muscle wasting varying across individual muscles,^6^ the extent of atrophy likely depends on the method of assessment and immobilization model used.^5^ For instance, while ~10% declines in fibre cross‐sectional area (CSA) were reported after 4 days of knee‐bracing,^29^ 5 days of leg‐casting showed no measurable decline in fibre CSA, despite ~3.5% decreases in whole muscle CSA.^4^ In the present study, using independent measurement techniques, we show significant declines in DXA derived thigh lean mass loss of ~1.7% which correlated with significant declines in VL MT of ~4%. Our data therefore agree with previous literature highlighting that muscle mass loss occurs rapidly at the onset of immobilization. Further, these results support the use of ultrasound as an indicator of muscle size^31^ at least in healthy individuals; the reduced invasiveness and cost offering a potential tool to increase measurement frequency during early atrophy.

Muscle mass is controlled by the balance between MPS and MPB. Earlier studies of MPS during immobilization utilized AA tracers representing the gold standard in assessing acute fasted‐state MPS and/or MPS responses to external stimuli (nutrition/inactivity etc.). These studies revealed that immobilization results in declines in both fasted and fed‐state MPS, that is, inducing a state of ‘anabolic resistance’.^9^, ^10^, ^32^ Despite calculations from these studies suggesting that declines in muscle mass may be entirely explained by declines in MPS,^10^ the very nature of acute intravenous AA tracer studies means they provide only a ‘snapshot’ of proteostasis.^33^ In contrast, the use of oral D_2_O provides an effective tool to quantify integrated MPS, capturing anabolic responses over the entire measurement period.^34^, ^35^ Here, we combined integrated D_2_O MPS measures along acute AA tracer approaches to capture both early changes in integrated MPS and acute feeding responses. We show that MPS is sustainably depressed over 4 days of immobilization and that this was correlated with loss of *VL* MT. Similarly, declines in MPS over 7 days of immobilization correlated with declines in quadriceps muscle volume determined by MRI. Together, these results provide good evidence that declines in MPS dominate the muscle atrophy process. Previous similar integrated measures of MPS have shown substantial declines over 14 and 7 days of immobilization,^28^, ^35^ with no significant changes over 2 days.^28^ As such, we have shown the earliest declines in integrated MPS to date. Further, we show that by Day 4, this is primarily a result of impaired feeding responses and thus reduced MPS in both the fasted and fed states (also the earliest observation of immobilization induced anabolic resistance albeit congruent with longer term results). Intriguingly, we also estimated similar declines in daily integrated MPS from both our D_2_O measures and our daily acute MPS when assuming that post‐absorptive periods represent two‐thirds of a diurnal cycle. These findings further substantiate that MPS alone can explain declines in muscle mass, in the absence of rises in ‘bulk’ MPB.

Increased markers of MPB during the early stages of immobilization have resulted in MPB having a proposed role in early disuse atrophy.^16^, ^36^ Previously, A‐V balance techniques showed no difference in whole‐body protein breakdown after 2 weeks of immobilization.^15^ Another study showed that with 21 days of bed rest, there was no increase in MPB.^37^ In contrast, indirect indicators of contractile protein breakdown (3‐methylhistidine) have been shown to be elevated during the early stages of immobilization (3 days)^38^; however, the validity of this result has been questioned^39^ given e.g. non‐skeletal muscle sources of 3‐methylhistidine. More recently, MPB was determined to be lower during a period of immobilization vs. re‐training,^40^ yet this study was not designed to compare with a control period, limiting conclusions that may be drawn. In contrast to measures of dynamic MPB, increased markers of MPB early with immobilization have been reported, most noticeably, those of ubiquitin proteasome pathway (e.g. MAFbx). However, eIF3f, a key initiation factor in protein synthesis, is a major target for MAFbx and subsequent degradation.^41^ As such, we posit that increased markers of MPB act to degrade key components of the protein synthetic machinery rather than bulk myofibrillar contractile elements. To determine the role of bulk MPB in muscle atrophy, we adopted a pulse‐chase tracer technique to assess dynamic rates of MPB^14^ in addition to applying robust extrapolations from the D_2_O tracer. Firstly, we found no differences in the rate of tracer decay between the immobilized vs. the control leg over 60 min in the fasted state, indicating MPB was not increased in the immobilized leg. Secondly, to assimilate MPB over the 4 day period, we combined rates of MPS with the net change in muscle mass per day (FGR) to calculate FBR. With the difficulties in accurately measuring FBR during atrophy/hypertrophy, these equations have shown to provide an accurate estimation of FBR in response to various interventions.^20^, ^42^, ^43^ Using these validated equations, we observed no increases in FBR during immobilization. Furthermore, changes in FBR did not correlate with declines in muscle mass, with no change in the expression of key markers of MPB. For that reason, our data fully support MPB having a minimal role in driving immobilization induced human muscle atrophy.

Accompanying dynamic measures of MPS and MPB, we quantified the phosphorylation and abundance of proteins involved in proteostasis. Despite decreased cumulative MPS, owing to deficits in fed (and perhaps fasted) state MPS, anabolic responses to feeding (P‐AKT^S473^ and P‐RPS6^S235/236^) were not reduced with immobilization. A lack of alignment between muscle anabolic signalling and fractional synthesis rate has been reported previously.^44^, ^45^ While providing valuable mechanistic insight, these measures represent a snapshot of intramuscular signalling, with peak responses likely occurring earlier in the feeding response.^44^ Furthermore, these samples were collected under a glucose clamp, with insulin signalling potentially swamping any effects of AA.^46^ Finally, we measured proteins that play a key role in both calcium activated and ubiquitin proteasome MPB pathways; agreeing with our measures showing a lack of increase in FBR or MPB, we observed no clear induction of intracellular signalling markers of MPB.

While adding novel insights into the effects of immobilization on muscle protein turnover at the onset of immobilization induced muscle atrophy, our study is not without limitations. With the paucity of data on rates of MPB in early immobilization, we calculated study power on previous reports of declines in post‐prandial MPS.^17^ Expectedly, we found consistent and robust declines in MPS, and using balance equations, we showed no change in FBR, despite a heightened numerical average over 4 days (notably, such calculations rely on accurate scan‐to‐scan measures over a short study period and utilize whole changes in TFFM resulting in increased variability). Given limitations of retrospective power calculations,^47^ we also report that effect sizes are far stronger for declines in MPS than in supporting any increase in FBR. Further supporting these data, we measured tracer decay rates within the VL, again, showing no change in MPB (although these dynamic measures of MPB were only performed at a single time point in the fasted stated and limited by subject number). Finally, we found only MPS and not FBR correlated with VL MT declines, with similar correlations having been independently reported by others.^28^

In conclusion, short‐term immobilization results in significant loss of skeletal muscle mass that is driven by, and which correlates with sustained declines in MPS. A lack of increase in acute tracer decay, markers of MPB, and calculated FBR suggest that bulk MPB has a minor, if any, role at the onset of leg immobilization induced muscle atrophy in humans; a time at which muscle is rapidly lost. We also demonstrate in agreement to previous work that decreased integrated MPS is accompanied by a rapid blunting of acute anabolic responses to hyperaminoacidemia and hyperinsulinemia, with acute fasted/fed MPS reflective of declines in 4 day integrated MPS. As such, we contend the development of effective ‘anti‐catabolic’ therapeutics should be targeted at enhancing basal MPS and anabolic responses to feeding.

## Funding

This work was supported by the Medical Research Council (grants MR/R502364/1 and MR/P021220/1) as part of the MRC‐ARUK Centre for Musculoskeletal Ageing Research awarded to the Universities of Nottingham and Birmingham, and the National Institute for Health Research, Nottingham Biomedical Research Centre. Dr S.M. Phillips reports grants from US National Dairy Council, during the conduct of the study; personal fees from US National Dairy Council, non‐financial support from Enhanced Recovery, outside the submitted work. In addition, Dr S.M. Phillips has a patent Canadian 3052324 issued to Exerkine and a patent US 20200230197 pending to Exerkine but reports no financial gains.

## Conflict of interest

None declared
